# Revealing the Giant Electromechanical Effects in Yttria‐Stabilized Zirconia Single Crystal

**DOI:** 10.1002/advs.76776

**Published:** 2026-07-21

**Authors:** Zhuwu Yi, Kai Pan, Chaoming Hu, Luocheng Liao, Zhijian He, Ziwei Guo, Yibao Wu, Changxing Zhao, Shuhong Xie

**Affiliations:** ^1^ Key Laboratory of Low Dimensional Materials and Application Technology of Ministry of Education School of Materials Science and Engineering Xiangtan University Xiangtan Hunan China; ^2^ Hunan Provincial Key Laboratory of Thin Film Materials and Devices School of Materials Science and Engineering Xiangtan University Xiangtan Hunan China

**Keywords:** atomic force microscopy, giant electrostriction, induced piezoelectricity, oxygen vacancies, yttria‐stabilized zirconia

## Abstract

Ionic conductors exhibiting giant electromechanical responses have emerged as promising alternatives to lead‐based relaxor ferroelectrics for high‐resolution actuators and sensors. However, the role of long‐range oxygen‐vacancy migration in governing electromechanical properties remains unclear and even controversial. Yttria‐stabilized zirconia single crystal (SC‐YSZ) provides an ideal model system to elucidate the origin of electrostriction and piezoelectricity with oxygen‐vacancy migration, owing to the absence of grain boundaries and substrate constraints. Here, atomic force microscopy (AFM) is employed to probe the electromechanical coupling responses of SC‐YSZ, displaying pronounced orientation‐dependent features and non‐intrinsicity in [100]‐, [110]‐, and [111]‐oriented SC‐YSZs. Notably, the giant electrostrictive coefficient |*M*_33_| of 5.82 × 10^−17^ m^2^ V^−2^ and remarkable pseudo‐piezoelectric coefficient |*d*_33_| of 218 pm V^−1^ are obtained at the frequency of 10 mHz in [100]‐oriented SC‐YSZ. Local relaxation measurements, macroscopic ionic conductivity, and first‐principles calculations reveal that the orientation‐dependent electrostrictive and pseudo‐piezoelectric responses arise from the long‐range migration of oxygen vacancies, governed by direction‐dependent migration difficulty under electric fields. This work provides direct evidence linking giant electromechanical responses with long‐range oxygen‐vacancy migration, and highlights that such effects in SC‐YSZ cannot be neglected when employed as substrates or composite layers in electroactive materials and device systems.

## Graphical abstract

Giant electromechanical responses are uncovered in yttria‐stabilized zirconia single crystal (SC‐YSZ), exhibiting pronounced orientation dependence. The giant electrostriction and piezoelectricity are directly correlated with the long‐range transport kinetics of oxygen vacancies. These findings not only establish vacancy‐mediated electromechanical coupling in ionic conductors but also suggest that SC‐YSZ requires careful consideration when used as substrates or epitaxial layers in electroactive devices.

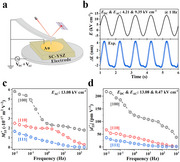

## 1 Introduction

Ionic conductors exhibit giant electrostriction in rare‐earth‐metal‐doped CeO_2_ [1, 2, 3, 4, 5], (Y, Nb)‐stabilized δ‐Bi_2_O_3_ [6, 7], LAMOX [8, 9, 10], with electrostrictive responses exceeding predictions based on Newnham's scaling law by more than two orders of magnitude recently [11]. The electrostrictive coefficient |*M*_xx_| reaches values above ∼ 10^−14^ m^2^ V^−2^, surpassing those of state‐of‐the‐art PMN‐ and PZN‐based ferroelectrics substantially [7]. Notably, this giant electrostriction gives rise to pronounced pseudo‐piezoelectricity (∼ 200 000 pm V^−1^), originating from symmetry breaking induced by the electric‐field‐driven redistribution of oxygen vacancies [12, 13]. These unexpectedly high electromechanical properties, compatible with silicon‐based fabrication processes, environmental benignity, and non‐toxic, render ionic conductors promising alternatives to lead‐based relaxor ferroelectrics for sensors, actuators, and memristive devices in micro/nano‐electromechanical systems [14, 15, 16].

The giant electrostrain is generally attributed to tunable elastic dipoles arising from the orientational migration of oxygen vacancies, which can be modulated by lattice distortions [1, 17], grain‐boundary barrier effect [3, 18], and lattice mismatch induced by substrates or interlayers [7, 19]. However, the influence of long‐range oxygen‐vacancy migration on electromechanical properties remains unclear and, in some cases, controversial. In polycrystalline ceria‐based ceramics, larger grain sizes are typically associated with higher ionic conductivity [18, 20, 21], whereas electrostriction has been reported to either increase [22] or decrease [3, 18, 23], depending on microstructural conditions. This non‐monotonic relationship between electrostriction and ionic conductivity obscures the role of grain boundaries in governing electrostrictive behavior [18]. In Gd‐doped ceria (CGO) single‐crystalline, the electrostrictive coefficient increases with decreasing temperature below room temperature, concomitant with reduced ionic or defect mobility [24]. Notably, the electrostrictive coefficient *M*_33_ of ∼ 10^−18^ m^2^ V^−2^ in SC‐CGO is substantially smaller than the value of ∼ 10^−16^ m^2^ V^−2^ reported for CGO ceramic [22, 24], further suggesting the unclear correlation between the electrostriction and long‐range oxygen‐vacancy migration.

Yttria‐stabilized zirconia single crystal (SC‐YSZ), which exhibits excellent thermal stability, superior mechanical properties, and high chemical stability, is widely utilized in solid oxide fuel cells [25, 26, 27, 28], photoelectronic devices [29, 30, 31], and silicon‐based semiconductor technologies [32, 33, 34, 35]. In the absence of grain boundaries, substrate‐induced constraints, and electrochemical reaction, bulk SC‐YSZ serves as an ideal model system to elucidate the correlation between electrostriction/pseudo‐piezoelectricity and oxygen‐vacancy migration under an electric field. Although YSZ bulk ceramics and thin films have been established to exhibit pronounced non‐classical electrostriction [12], yet the electrostrictive and pseudo‐piezoelectric responses of SC‐YSZ remain largely unexplored. Here, strain‐based atomic force microscopy (AFM) is employed to probe the electromechanical coupling responses of [100]‐, [110]‐, and [111]‐oriented SC‐YSZs and to explore their dependence on applied electric fields. Under combined DC and AC electric field excitation at low frequencies, [100]‐oriented SC‐YSZ exhibits both giant electrostriction and remarkable pseudo‐piezoelectricity, comparable to those of state‐of‐the‐art lead‐containing piezoelectrics. A direct relationship between these pronounced electromechanical effects and the long‐range transport kinetics of oxygen vacancies is established in SC‐YSZ ionic conductors through relaxation measurements, impedance analysis, and first‐principles calculations. This study not only demonstrates large electrostriction and pseudo‐piezoelectricity in SC‐YSZs but also highlights the need for caution when employing SC‐YSZ as a substrate or epitaxial layer in electroactive materials and device systems.

## 2 Results and Discussion

**FIGURE 1:**
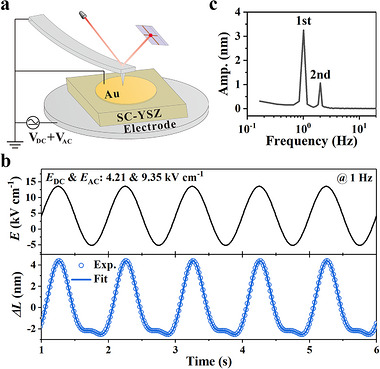
The electromechanical responses in [100]‐oriented SC‐YSZ. (a) The schematic illustration of AFM measurement. (b) The time‐resolved electromechanical displacement response (blue circle) under *E*_DC_ = 4.21 kV cm^−1^ and *E*_AC_ = 9.35 kV cm^−1^ at 1 Hz, which is fitted by a Fourier sine series (blue line). (c) The FFT resolved amplitude of the displacement response in Figure 1b.

The displacement responses of cubic fluorite structure SC‐YSZs (Y_2_O_3_: ZrO_2_ = 8: 92 mol%, Figure S1a) with an average thickness of 107 µm were characterized via atomic force microscopy (AFM) as depicted in Figure 1a. The top surface of SC‐YSZ was coated by a large Au electrode (radius 2 mm), and its bottom surface was affixed to a conductive iron plate using silver paste. The configuration ensures the equipotential condition between the conductive AFM probe and Au electrode, thereby minimizing the electrostatic effects [36]. DC/AC voltages were applied to the bottom electrode with the top electrode grounded, given that the electrostatic field underneath the top electrode is assumed to be uniform in SC‐YSZ (Section S1). And the SC‐YSZs were polished to minimize topographic interference (Figure S1b). As shown in Figure 1b, the thickness change *ΔL* (blue circles) of the [100] oriented SC‐YSZ was measured under a DC electric field with *E*_DC_ = 4.21 kV cm^−1^ and an AC electrical field with *E*_AC_ = 9.35 kV cm^−1^ at a frequency of 1 Hz. Here, *ΔL* can be fitted by using the expansion of the Fourier sine series $\Delta L=\sum_{n=1}L_{n}\sin\left(2n\pi ft-\varphi_{n}\right)$, where *L_n_* and *φ_n_* are the nth order of thickness change and phase lag, *f* and *t* are the frequency and time, respectively [12]. The superposed displacement response was further confirmed to comprise the first‐ and second‐order harmonic components through fast Fourier transform (FFT) analysis, as shown in Figure 1c. The second‐order response arises from electrostriction, whereas the first‐order response may stem from a combination of piezoelectricity and the Vegard effect. When subjected to a 3 V AC voltage at the resonance frequency of ∼ 341 kHz, SC‐YSZ showed no intrinsic piezoelectric response consistent owing to its cubic crystalline structure, with the minor Vegard strain associated with chemical expansion (Figure S1c,d). However, the first‐order displacement response in Figure 1c is substantially larger than that of measured under *E*_AC_ alone (Figure S4), indicating that the electromechanical response may be governed by the migration dynamics of oxygen vacancies in SC‐YSZ, reminiscent of behaviors reported in Gd‐doped ceria and related ion conductors [12]. The first‐order displacement response in SC‐YSZ is attributed to the pseudo‐piezoelectric response, whereby the applied DC electric field breaks the symmetry charge distribution [12, 37, 38] and consequently induces polarization.

**FIGURE 2:**
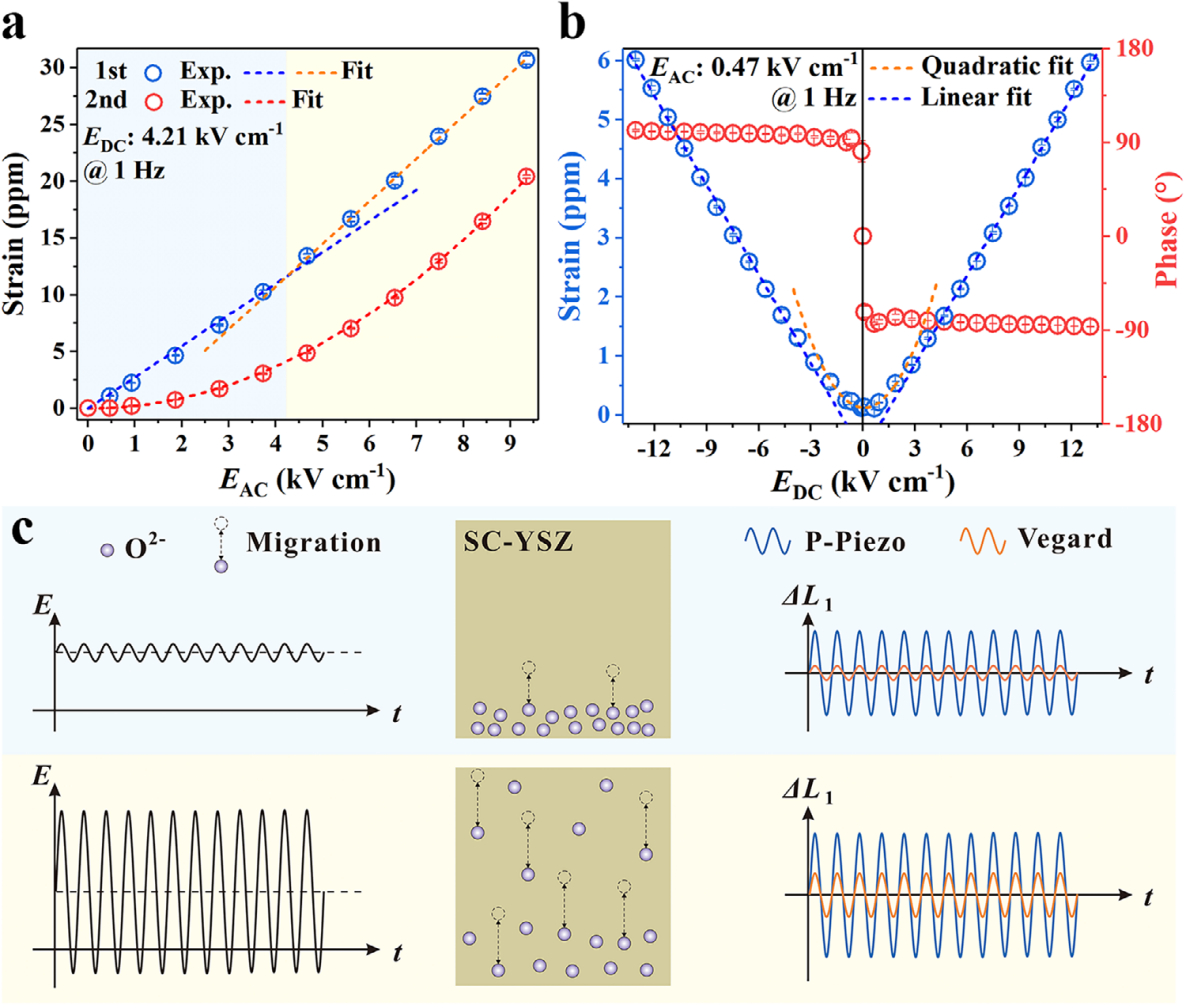
The electromechanical responses under different *E*_AC_ or *E*_DC_ in [100]‐oriented SC‐YSZ. (a) The first and second‐order electromechanical as a function of *E*_AC_ at 1 Hz, measured at *E*_DC_ = 4.21 kV cm^−1^. (b) The amplitude and phase angle of strain under different *E*_DC_ and a constant *E*_AC_ = 0.47 kV cm^−1^ at 1 Hz. (c) The schematic of the first‐order displacement *ΔL*_1_ under different *E*_AC_ and a fixed *E*_DC_.

To examine the influences of *E*_AC_ on the electromechanical response in SC‐YSZ, we fixed the *E*_DC_ at 4.21 kV cm^−1^ and varied *E*_AC_ from 0 to 9.5 kV cm^−1^ at 1 Hz, as illustrated in Figure 2a. The resolved second‐order electromechanical (red circles) can be fitted well by a parabola function (red dashed line), exhibiting an obvious electrostriction. The fitted electrostriction coefficient is approximately 2.31 × 10^−17^ m^2^ V^−2^, in line with the commercial relaxor ferroelectric materials [2, 24, 39]. However, the correlation between the first‐order electromechanical responses and *E*_AC_ cannot be fitted by a single line. A slight jump in slope appears as *E*_AC_ exceeds *E*_DC_, indicating that the first‐order electromechanical response can be perturbed when the electromechanical response varies linearly with *E*_DC_ as plotted in Figure 2b. This result demonstrates that the overall response is dominated by the first‐order harmonic component. A 180° phase shift emerges in the electromechanical response when the direction of *E*_DC_ is reversed from upward to downward, indicating that the first‐order electromechanical at large *E*_DC_ cannot be primarily ascribed to the Vegard effect. While it is worth noting that a quadratic fit can still capture the electromechanical responses at lower *E*_DC_, which is consistent with the trend in Figure 2a.

To clearly analyze the electromechanical responses under electric field stimuli, we consider the total electrostrain *S* to arise from both Vegard effect and electrostriction interpreted by [12, 40]

(1) $$S=\beta \delta c+ME^{2}$$

where *β* and *M* are the Vegard and electrostriction coefficients, respectively; *δc* is the concentration change of oxygen vacancies, and *E* is the applied electric field. Here, the Vegard effect is attributed to the first‐order electromechanical response as in previous studies [40, 41]. Therefore, the electrostrain can be further expanded as follows.

(2) $$\begin{array}{ccc} S & = & \tilde{\beta }E+ME^{2}=ME_{AC}^{2}\sin^{2}\left(ft+\varphi \right)\\ & & +\left(\tilde{\beta }+2ME_{DC}\right)E_{AC}\sin\left(ft+\varphi \right)+\left(\tilde{\beta }E_{DC}+ME_{DC}^{2}\right) \end{array}$$

with the coefficient $\tilde{\beta }$ relating to the Vegard effect. The oxygen vacancies are nearly frozen when the *E*_DC_ is much larger than the *E*_AC_, resulting in a small Vegard strain, as shown in Figure 2c. Consequently, the first‐order displacement *ΔL*_1_ is dominated by the induced pseudo‐piezoelectric (P‐Piezo) response, indicating that the application of *E*_DC_ can activate a piezoelectric‐like behavior in SC‐YSZ [12]. As *E*_AC_ increases to values comparable to or exceeding *E*_DC_, a larger population of oxygen vacancies begins to move up and down, thereby enhancing the Vegard strain *βδc*. In contrast, the P‐Piezo coefficient remains nearly constant because the electrostriction coefficient is field‐invariant (Figure 2a). Consequently, the increasing number of mobile oxygen vacancies is likely responsible for the rise in the slope of the first‐order strain vs. *E*_AC_ in Figure 2a. It should also be noted that the Vegard‐derived first‐order electromechanical response is smaller than the pseudo‐piezoelectric contribution substantially, owing to the very small Vegard coefficient, as confirmed in Figure S4.

To analyze the electromechanical responses quantitatively, the effective electrostriction coefficient $M_{33}^{*}$ and effective pseudo‐piezoelectric coefficient $d_{33}^{*}$ are defined as follows [12].

(3) $$M_{33}^{*}=\frac{\Delta L_{2}}{L}E_{AC}^{-2}$$

(4) $$d_{33}^{*}=\frac{\Delta L_{1}}{L}E_{AC}^{-1}=\tilde{\beta }+2M_{33}^{*}E_{DC}$$

where *L* is the thickness, *ΔL*_1_ and *ΔL*_2_ are the first‐ and second‐order of thickness changes, respectively.

**FIGURE 3:**
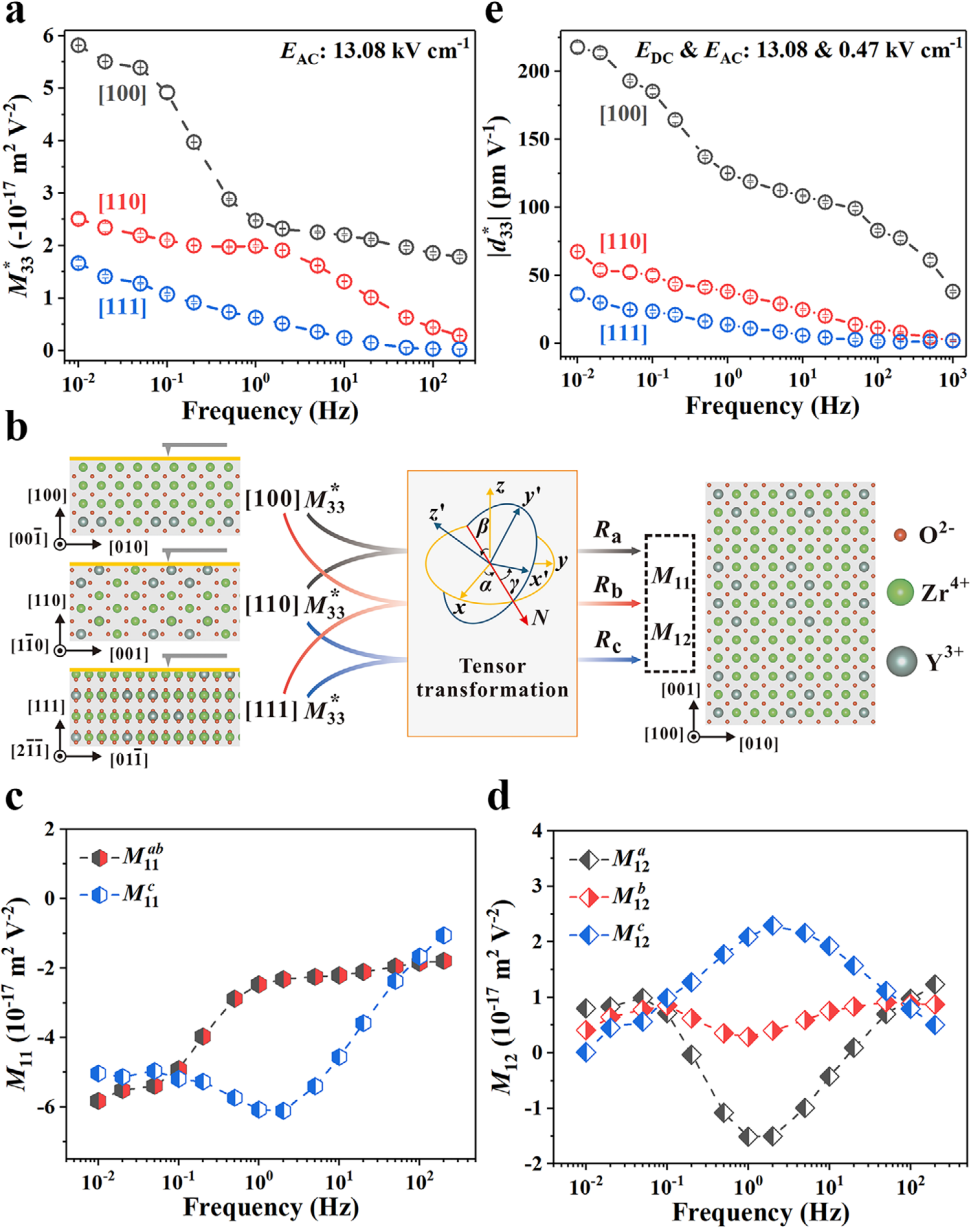
The effective electrostrictive and pseudo‐piezoelectric coefficients in [100]‐, [110]‐, and [111]‐oriented SC‐YSZ samples. (a) The effective electrostriction coefficients $M_{33}^{*}$ under *E*_AC_ = 13.08 kV cm^−1^ at different frequencies. (b) The schematic of resolving the intrinsic electrostrictive coefficient *M_ij_* by tensor transformation. (c) The resolved intrinsic electrostriction coefficients *M*_11_ and (d) *M*_12_ under different frequencies of *E*_AC_. (e) The magnitude of effective pseudo‐piezoelectric coefficients |$d_{33}^{*}$| under the superposition of *E*_DC_ & *E*_AC_ at different frequencies.

We examined the effective electrostriction coefficients $M_{33}^{*}$ of SC‐YSZs oriented along [100], [110], and [111] axes under AC electric fields at various frequencies, as shown in Figure 3a. The effective electrostrictive coefficients were confirmed to be negative based on the phase reversal when the DC bias switched from positive to negative (Section S2). For all orientations, the effective electrostrictive coefficients decrease with increasing frequency, consistent with the characteristic frequency‐relaxation behavior in ceria‐based materials [3, 22, 42]. Notably, $|M_{33}^{*}|$ is the largest for the [100]‐oriented SC‐YSZ and the smallest for the [111]‐oriented SC‐YSZ, revealing a pronounced orientation‐dependent electrostriction effect. In particular, the [100]‐oriented SC‐YSZ exhibits a giant $|M_{33}^{*}|$ of 5.82 × 10^−17^ m^2^ V^−2^ at 10 mHz, approaching the values reported for rare‐earth‐doped ceria ceramics [3, 22, 42]. This orientation dependence becomes especially prominent at low frequencies (< 0.1 Hz), consistent with the dynamic migration of the oxygen vacancies.

The origin of effective electrostriction is typically attributed to the crystal lattices in piezoelectrics and ferroelectrics, commonly referred to as intrinsic electrostriction. In defect‐rich fluorite materials, such as ceria‐based ceramics, δ‐Bi_2_O_3_, however, the effective electrostriction also contains a substantial induced contribution associated with the migration of oxygen vacancies. To investigate the correlation between the effective and intrinsic electrostriction effects, we treated SC‐YSZs as a cubic structure and resolved the intrinsic electrostrictive coefficients of [001]‐oriented SC‐YSZ by applying tensor‐rotation transformations [43, 44, 45] (routes *R*_a_‐*R*_c_) from two of the three measured orientations as illustrated in Figure 3b. The details of the tensor‐rotation transformation procedure are provided in Section S3. As shown in Figure 3c,d, the resolved *M*_11_ and *M*_12_ through the three routes exhibit no consistent trend, and even the signs of the intrinsic electrostrictive coefficients cannot be determined reliably. These results suggest that the measured electrostriction is dominated by induced electrostriction instead of intrinsic electrostriction associated with their pristine lattice structures in the absence of an electric field.

The orientation‐dependent electrostriction results in an orientation‐dependent effective pseudo‐piezoelectricity under the superposed *E*_DC_ = 13.08 kV cm^−1^ and *E*_AC_ = 0.47 kV cm^−1^, as illustrated in Figure 3e. Under a DC electric field, the oxygen ions and vacancies are driven to separate [12, 37, 38], resulting in long‐range dipoles that produce an induced polarization in SC‐YSZ, thereby generating pseudo‐piezoelectricity in cubic SC‐YSZ. The orientation‐dependent effective pseudo‐piezoelectricity likely originates from the anisotropic mobility of the oxygen vacancies along different crystallographic orientations. Furthermore, the effective pseudo‐piezoelectric coefficients decrease with increasing frequencies, which is consistent with the frequency dependence of |$M_{33}^{*}$|, as expected for vacancy‐migration‐dominated processes. Remarkably, the [100]‐oriented SC‐YSZ exhibits a maximum $|d_{33}^{*}|$ value of 218 pm V^−1^ at 10 mHz, comparable to that of widely used Pb(Zr,Ti)O_3_ (PZT) ceramics [46, 47, 48]. In comparison with the YSZ ceramic and film, the SC‐YSZ is expected to exhibit superior performance at the same electric field, as shown in Table S1. These findings suggest that the large electromechanical responses of SC‐YSZ cannot be negligible under electrical loadings, which is important to consider when employing SC‐YSZ as substrates or epitaxial layers in electromechanical systems.

**FIGURE 4:**
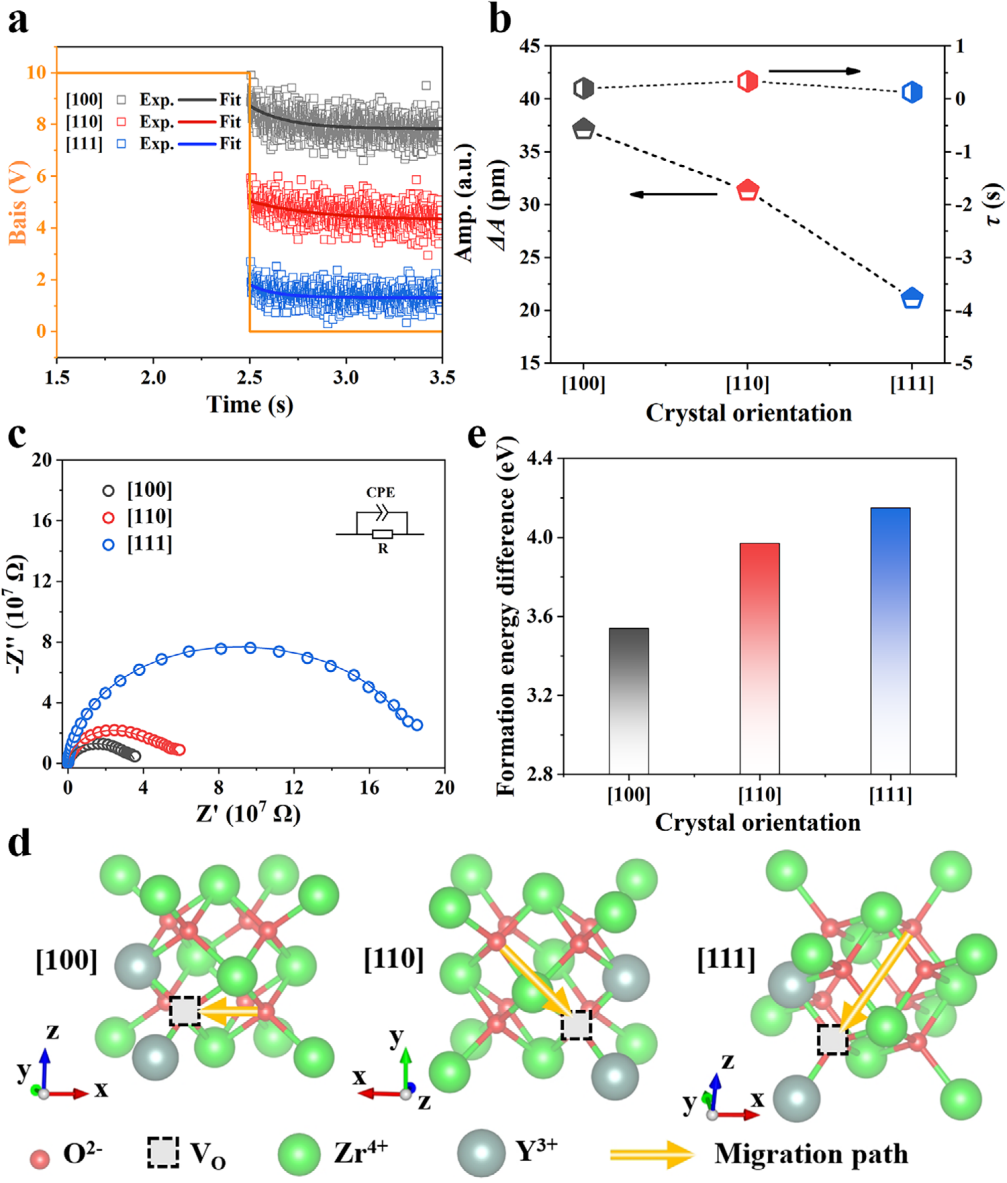
Dynamics of oxygen vacancies in [100]‐, [110]‐, and [111]‐oriented SC‐YSZ samples. (a) Local relaxation after applying +10 V as a function of time under AFM experiment. (b) The fitted relaxation parameters in Figure 4a. (c) The Nyquist plots of SC‐YSZs at room temperature. (d) The schematic of the migration path of oxygen vacancies in SC‐YSZs. (e) The formation energy difference of the oxygen vacancies along the [100], [110], and [111] migration paths.

To reveal the correlation between the electromechanical responses and dynamics of the oxygen ions or vacancies, the relaxation and impedance analyses have been performed on [100]‐, [110]‐, and [111]‐oriented SC‐YSZ samples. During the relaxation experiment, we applied a positive 10 V DC step voltage, along with a 3 V AC drive voltage to the conductive AFM probe. The subsequent relaxation of the signal was then measured when the DC voltage was removed, as shown in Figure 4a. It is observed that the electromechanical response amplitude increases with the application of a positive DC voltage, and then returns to the baseline after the DC bias is removed due to the redistribution of the oxygen ions and vacancies underneath the probe [49]. In particular, the relaxation curves can be fitted by *A* = *A*_0_ + *A*_1_exp (− *t*/τ) [50], where *τ* is the time constant, and *A*, *A*_0_, and *A*_1_ are the measured signal, the static response at equilibrium, and the relaxation amplitude, respectively. And the fitted parameters are shown in Figure 4b, wherein the magnitude of the response amplitude depends on the crystallographic orientation, showing a maximum value of *ΔA* = 37.14 pm along the [100]‐oriented SC‐YSZ, followed by [110] and then [111], exhibiting the same trend as that of |$M_{33}^{*}$| and |$d_{33}^{*}$|. This local relaxation suggests that the electromechanical of SC‐YSZs strongly links to the local ionic relaxation. Nyquist plots of the three oriented SC‐YSZs at room temperature are exhibited in Figure 4c, illustrating the mobility of the ionic migration at the macroscale [18, 51]. Among the three oriented SC‐YSZ samples, the [100]‐oriented SC‐YSZ exhibits the lowest impedance and then followed by the [110]‐oriented SC‐YSZ, while the [111]‐oriented SC‐YSZ has the highest impedance. The orientation‐dependent ionic conductivity suggests that oxygen vacancies might be the easiest to migrate along the [100] yet the hardest along the [111] in SC‐YSZ.

To further verify the mobility of the oxygen vacancies along different directions at the atomic scale, the migration energies of oxygen vacancies were calculated by the first‐principles calculation in three oriented SC‐YSZ. Owing to the migration of oxygen vacancies depends strongly on the configurations of the neighboring cations around the diffusing oxygen vacancy, the oxygen vacancy migration energy is calculated by the formation energy difference of two configurations between the initial configuration (before migration) and the final configuration (after migration) [52]. As shown in Figure 4d,e, the formation energy difference of oxygen vacancies is the lowest in [100] and followed by [110], yet the highest in [111]‐oriented SC‐YSZ, agreeing well with the relaxation and impedance results. It further ensures that the mobility of oxygen vacancies has a strong relation with the electrostriction. However, there is no existence of the strain with the phase transition [12] under electric stimuli, as shown in Figure S7. Because only a slight shift of the diffraction peak to a higher angle under a large DC voltage of 1000 V was observed through in situ x‐ray diffraction (XRD) measurements, indicating a tiny decrease in lattice parameters. Actually, the SC‐YSZ is highly stable at room temperature, while the phase transition can occur with the elevating temperature higher than 550°C [53]. Under an AC electric field, the long‐range migration of oxygen vacancies can induce the lattice distortion, thereby generating a giant electrostriction [1, 22, 54]. After applying a large *E*_DC_, the oxygen vacancies migrated and then nearly frozen to maintain the electrical dipoles in SC‐YSZ for the generation of both giant electrostriction and pseudo‐piezoelectric effects under small *E*_AC_ stimuli.

## 3 Conclusions

In summary, the electromechanical responses exhibit first‐ and second‐order responses under the mixed excitation of AC and DC electric fields in SC‐YSZ via the AFM experiment. The maximum magnitude of electrostrictive coefficient |*M*_33_| approaches 5.82 × 10^−17^ m^2^ V^−2^ and pseudo‐piezoelectric coefficient |*d*_33_| reaches 218 pm V^−1^ at the frequency of 10 mHz in [100]‐oriented SC‐YSZ. The orientation‐dependent electrostriction effect leads to a similar trend of orientation‐dependent pseudo‐piezoelectricity, wherein the largest effective electromechanical effect was observed in [100]‐oriented SC‐YSZ, followed by [110], and then the smallest is along [111] direction. The intrinsic electrostriction coefficients of [001] oriented SC‐YSZ have been resolved using the tensor transformation, while the results show that the effective electrostriction coefficient does not originate primarily from the intrinsic electrostriction of the crystal lattice, but arises from the effective electrostriction induced by oxygen vacancies. The local relaxation and macroscale ionic conductivity show that the orientation‐dependent electrostriction coefficients and pseudo‐piezoelectric coefficients of SC‐YSZ are owing to the long‐range migration of the oxygen vacancies influenced by the migration difficulty in different directions under the electric field. The giant electromechanical responses were suggested from the defect‐related dipoles after the long‐range migration of the oxygen vacancies. Our study points out that the giant electromechanical effects of the SC‐YSZ cannot be ignored for applications of SC‐YSZ as substrates and composite layers in electromechanical materials and devices.

## 4 Experimental Section

The polished [100]‐, [110]‐, and [111]‐oriented SC‐YSZ samples were purchased from HeFei Crystal Technical Material Co. Ltd. The SC‐YSZ samples were pasted on an iron conductive sheet with silver paste. A thin circular mask plate was used to prepare Au electrode on the top surface of SC‐YSZ samples by plasma sputtering coating (Hefei Kejing Materials Technology, VTC‐16‐3HD). The electromechanical responses of SC‐YSZ samples were measured through the experimental setup based on atomic force microscopy (AFM, Asylum Research, MFP‐3D Infinity). During the AFM experiment, displacement responses were recorded at 3∼5 randomly selected points on the top electrode. The electrochemical impedance spectroscopy (EIS) measurement was conducted using an electrochemical workstation (CorrTestTM, CS2350H). The open‐circuit voltage was approximately 5 mV without DC bias. The x‐ray diffraction (XRD) patterns were acquired with a Rigaku Smartlab, and the voltages of in situ XRD were applied with a function signal generator (Rigol, DG1062Z) and a voltage amplifier (Trek, 610C).

## Author Contributions

**Kai Pan**: conceptualization, investigation, writing – original draft, methodology, visualization, writing – review and editing, project administration, formal analysis, supervision, resources, funding acquisition. **Zhuwu Yi**: conceptualization, investigation, writing – original draft, methodology, writing – review and editing, visualization, validation, data curation. **Changxing Zhao**: investigation. **Shuhong Xie**: investigation, funding acquisition, supervision, resources, writing – review and editing, formal analysis, project administration. **Chaoming Hu**: software, investigation. **Ziwei Guo**: investigation. **Luocheng Liao**: methodology, investigation. **Yibao Wu**: investigation. **Zhijian He**: software, investigation.

## Funding

This work was supported by the National Natural Science Foundation of China (12272333, 12572182), the Key Project of Natural Science Foundation of Hunan Province (2025JJ30004), and the Open Project Program of Key Laboratory of Low Dimensional Materials & Application Technology (Xiangtan University), Ministry of Education, China (2024YB01).

## Conflicts of Interest

The authors declare no conflicts of interest.

## Supporting information




**Supporting File**: advs76776‐sup‐0001‐SuppMat.doc.

## Data Availability

The data that support the findings of this study are available from the corresponding author upon reasonable request.
