# Supplementary material for: Revealing the Giant Electromechanical Effects in Yttria‐Stabilized Zirconia Single Crystal
Source: Adv Sci (Weinh). 2026 Jul 21:e76776. Online ahead of print. doi: 10.1002/advs.76776 (PMC13387036; doi:10.1002/advs.76776)
Supplement: Supplementary file 1 — Supporting File: advs76776‐sup‐0001‐SuppMat.doc. [file ADVS-9999-e76776-s001.doc]

Supporting Information

Revealing the Giant Electromechanical Effects in Yttria-Stabilized Zirconia Single Crystal

Zhuwu Yi, Kai Pan*, Chaoming Hu, Luocheng Liao, Zhijian He, Ziwei Guo, Yibao Wu, Changxing Zhao, Shuhong Xie*

**S1. The finite element simulations of the electric potential and electric field distribution**

To determine the distributions of the potential and electric field in an yttria-stabilized zirconia single crystal (SC-YSZ) during electromechanical measurement, a three-dimensional finite element simulation model was constructed as shown in Figure S2(a) and S2(b). The SC-YSZ was assumed to be surrounded by air and had dimensions of 5×5×0.1 mm, consistent with the atomic force microscopy (AFM) experiment. The circular top electrode with a radius of 2 mm was set to ground, while an electric potential *Φ* of 100 V was applied on the bottom electrode of the SC-YSZ. The material parameters of SC-YSZ were defined with a density of 5800 kg m^-3^, relative dielectric constant of 38, Young’s modulus of 205 GPa, and Poisson’s ratio of 0.31, respectively.^[1]^ As shown in Figure S2(c) and S2(d), the potentials are approximated to be uniform at a given depth underneath the top surface of the SC-YSZ, resulting in the uniform in-plane and out-of-plane electric fields, as further depicted in Figure S3.

**S2. The sign of the electrostriction coefficients**

To determine the sign of the electrostriction coefficients, switching spectroscopy piezo force microscopy (SS-PFM) measurement was conducted on SC-YSZ samples with three different crystallographic orientations under a DC bias. The amplitude and phase signals were acquired under “on-field” conditions. As shown in Figure S5, the [100]-, [110]-, and [111]-oriented SC-YSZs show nearly identical amplitude and phase at a resonance frequency of ~341 kHz. The phase results indicate that SC-YSZs exhibit the negative signs for their electrostriction coefficients,^[2]^ irrespective of crystallographic orientations.

**S3. The tensor-rotation transformation of electrostriction coefficients**

For cubic SC-YSZ, the electrostrictive tensor $M_{ijkl}^{*}$ for arbitrary crystallographic orientation can be obtained by transforming the electrostrictive tensor *M_ijkl_* of the [001]-oriented crystal using the tensor-rotation transformation.^[3,4]^

$M_{ijkl}^{*}=M_{ijkl}T_{ijkl}$, (S1)

where *T_ijkl_* is the fourth-order tensor, can be expanded by the second-order tensor *G_ij_* as^[5]^

$T_{ijkl}=\sum_{m=1}^{3} G_{im}G_{jm}G_{km}G_{lm}(\{i,j,k,l\}\in\{1,2,3\})$, (S2)

with **G** definded by the function of three Euler angles

. (S3)

For [100]-, [110]-, and [111]-oriented SC-YSZs, the rotation diagram from crystal coordinate to global coordinate can be presented in Figure S6, and the transformation matrices **G** of [100]-, [110]-, and [111]-oriented SC-YSZ are thus written as follows.

$G^{[100]}=\left[\begin{matrix} 0 & 0 & -1 \\ 0 & 1 & 0 \\ 1 & 0 & 0 \end{matrix}\right]$, $G^{[110]}=\left[\begin{matrix} \frac{1}{\sqrt{2}} & -\frac{1}{\sqrt{2}} & 0 \\ 0 & 0 & -1 \\ \frac{1}{\sqrt{2}} & \frac{1}{\sqrt{2}} & 0 \end{matrix}\right]$, $G^{[111]}=\left[\begin{matrix} \frac{2}{\sqrt{6}} & \frac{1}{\sqrt{6}} & \frac{1}{\sqrt{6}} \\ 0 & \frac{1}{\sqrt{2}} & -\frac{1}{\sqrt{2}} \\ -\frac{1}{\sqrt{3}} & \frac{1}{\sqrt{3}} & \frac{1}{\sqrt{3}} \end{matrix}\right]$. (S4)

The electrostriction coefficients $M_{ijkl}^{*}$ of [100]-, [110]-, and [111]-oriented SC-YSZ at the global coordinate are deduced as

$M_{[100]}^{*}=\left[\begin{matrix} M_{11} & M_{12} & M_{12} & 0 & 0 & 0 \\ M_{12} & M_{11} & M_{12} & 0 & 0 & 0 \\ M_{12} & M_{12} & M_{11} & 0 & 0 & 0 \\ 0 & 0 & 0 & M_{44} & 0 & 0 \\ 0 & 0 & 0 & 0 & M_{44} & 0 \\ 0 & 0 & 0 & 0 & 0 & M_{44} \end{matrix}\right]$, (S5)

$M_{[110]}^{*}=\left[\begin{matrix} \frac{M_{11}}{2}+\frac{M_{12}}{2} & M_{12} & \frac{M_{11}}{2}+\frac{M_{12}}{2} & 0 & 0 & 0 \\ M_{12} & M_{11} & M_{12} & 0 & 0 & 0 \\ \frac{M_{11}}{2}+\frac{M_{12}}{2} & M_{12} & \frac{M_{11}}{2}+\frac{M_{12}}{2} & 0 & 0 & 0 \\ 0 & 0 & 0 & 0 & 0 & 0 \\ 0 & 0 & 0 & 0 & \frac{M_{44}}{2} & 0 \\ 0 & 0 & 0 & 0 & 0 & 0 \end{matrix}\right]$, (S6)

$M_{[111]}^{*}=\left[\begin{matrix} \frac{M_{11}}{2}+\frac{M_{12}}{2} & \frac{M_{11}}{6}+\frac{5M_{12}}{6} & \frac{M_{11}}{3}+\frac{2M_{12}}{3} & 0 & \frac{\sqrt{2}M_{12}}{6}-\frac{\sqrt{2}M_{11}}{6} & 0 \\ \frac{M_{11}}{6}+\frac{5M_{12}}{6} & \frac{M_{11}}{2}+\frac{M_{12}}{2} & \frac{M_{11}}{3}+\frac{2M_{12}}{3} & 0 & \frac{\sqrt{2}M_{11}}{6}-\frac{\sqrt{2}M_{12}}{6} & 0 \\ \frac{M_{11}}{3}+\frac{2M_{12}}{3} & \frac{M_{11}}{3}+\frac{2M_{12}}{3} & \frac{M_{11}}{3}+\frac{2M_{12}}{3} & 0 & 0 & 0 \\ 0 & 0 & 0 & \frac{M_{44}}{3} & 0 & \frac{\sqrt{2}M_{44}}{6} \\ -\frac{\sqrt{2}M_{44}}{6} & \frac{\sqrt{2}M_{44}}{6} & 0 & 0 & \frac{M_{44}}{3} & 0 \\ 0 & 0 & 0 & \frac{\sqrt{2}M_{44}}{6} & 0 & \frac{M_{44}}{6} \end{matrix}\right]$. (S7)

According to Equations S5-S7, the electrostrictive coefficient $M_{33}^{*}$ equals to $M_{11}$, $(M_{11}+M_{12})/2$, and $(M_{11}+2M_{12})/3$ for [100]-, [110]-, and [111]-oriented SC-YSZ, respectively.


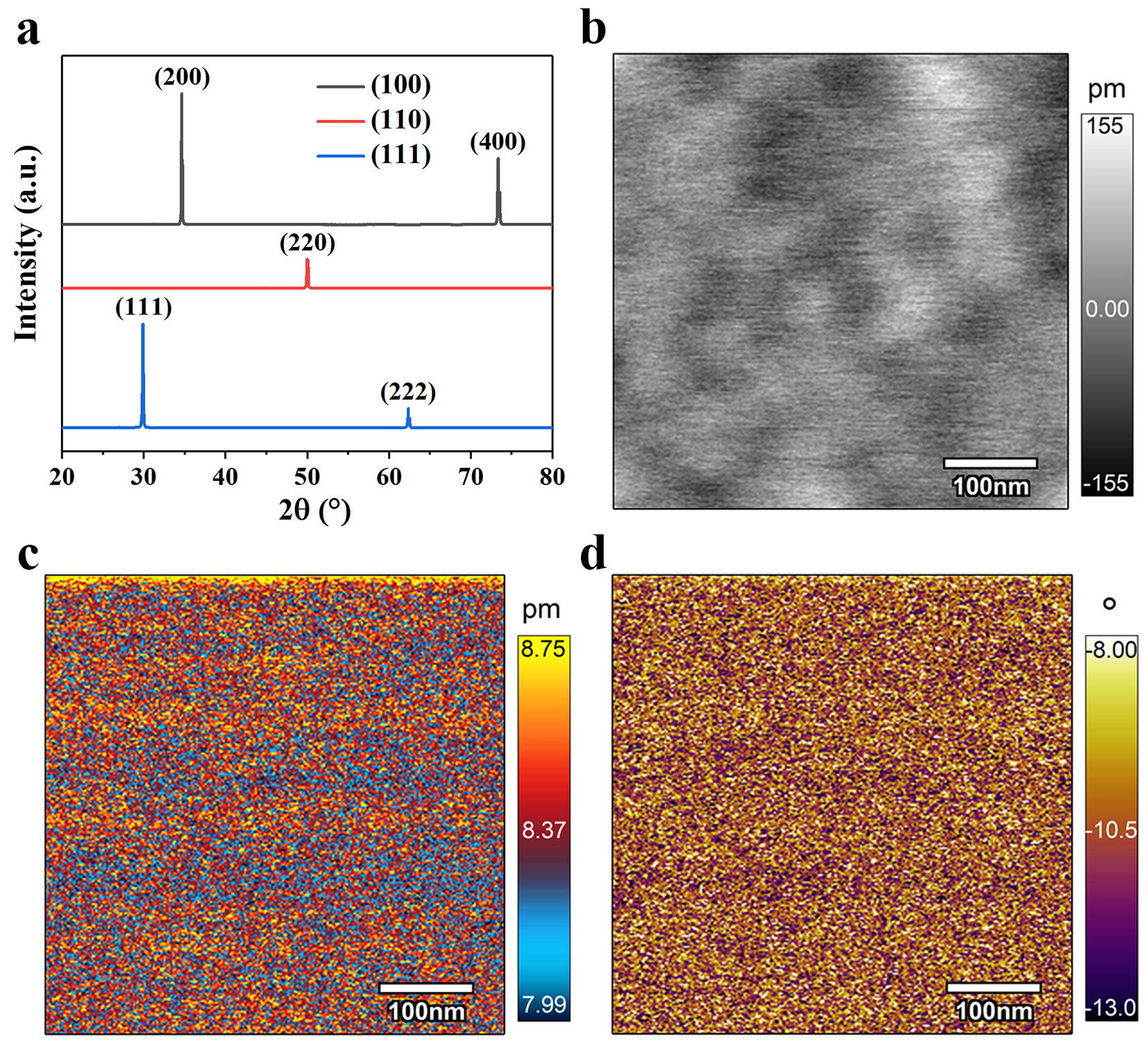


Figure S1. The structures and atomic force microscopy (AFM) images of SC-YSZ samples. (a) The X-ray diffraction (XRD) patterns of SC-YSZ samples. The (b) topography, (c) amplitude, and (d) phase scanned on the surface of [100]-oriented SC-YSZ within an area of 0.25 μm^2^ under an AC voltage of 3 V.


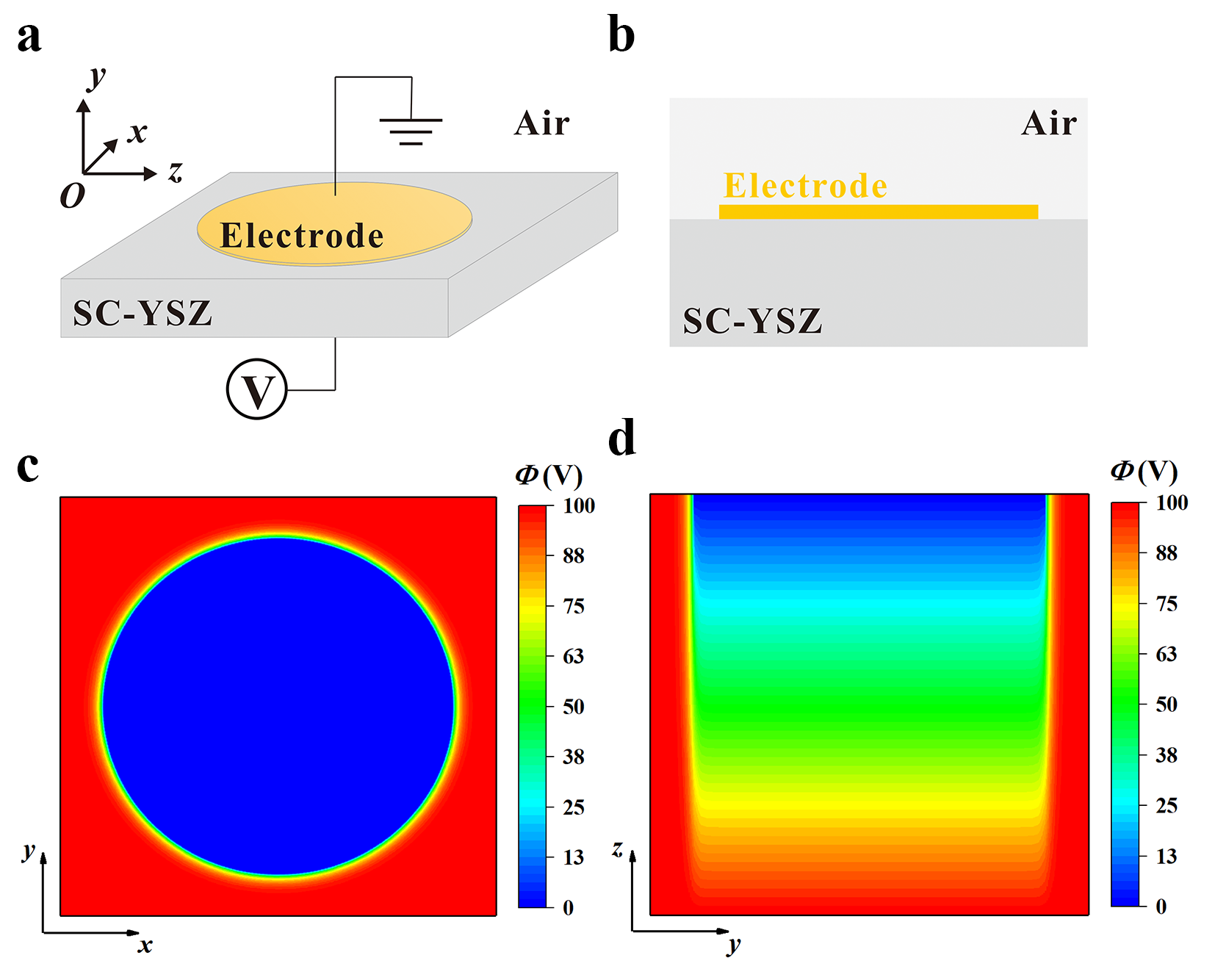


Figure S2. The (a) three-dimensional and (b) two-dimensional (front view) schematic diagram of a finite element simulation model. The distributions of electric potential by applying a DC voltage of 100 V in SC-YSZ through finite element simulation. The electric potential on (c) surface and in (d) cross-section.


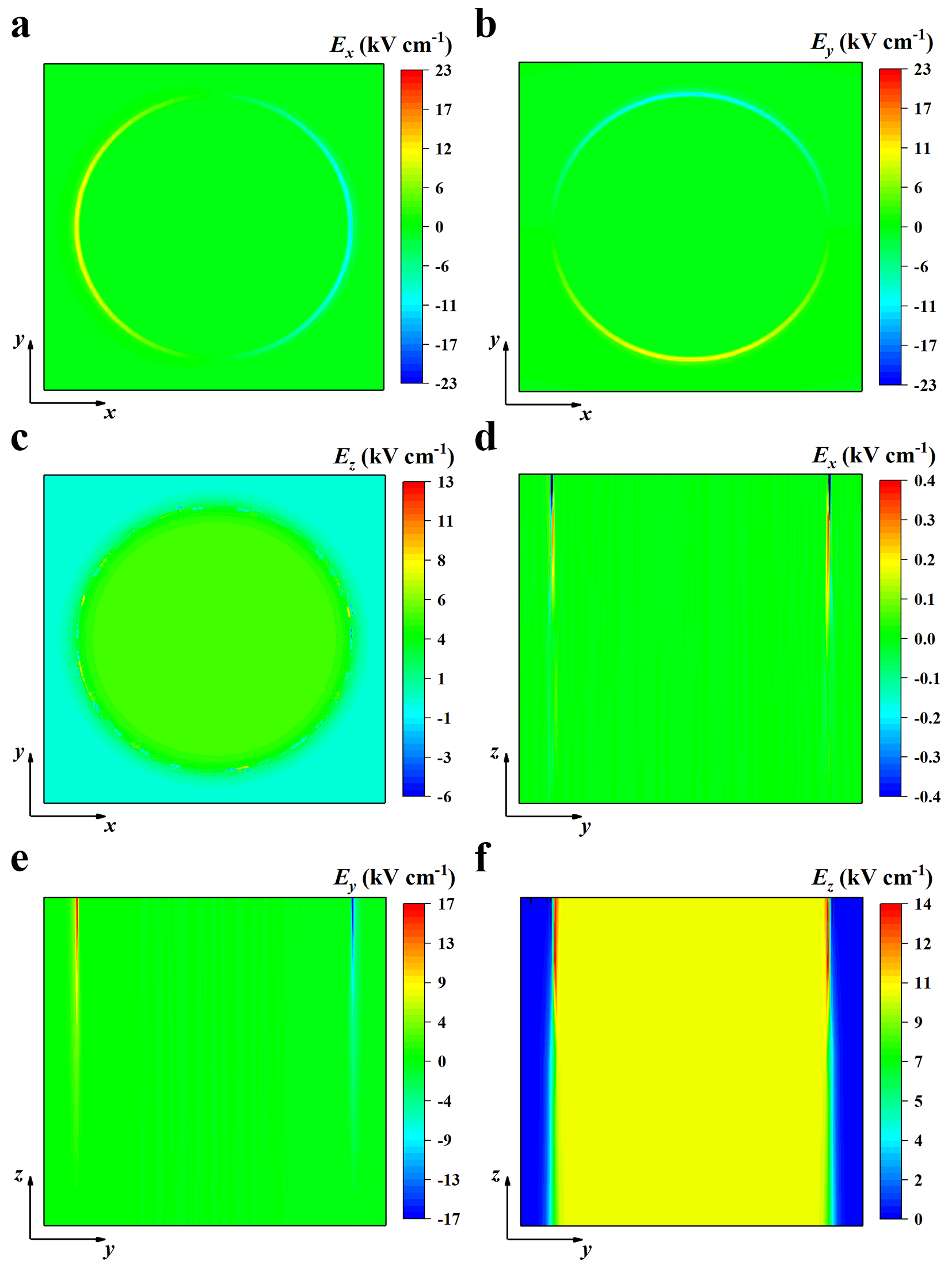


Figure S3. The distributions of the electric fields induced by applying a DC voltage of 100 V in SC-YSZ through the finite element simulations. The in-plane electric fields *E_x_*, *E_y_*, and out-of-plane electric field *E_z_* on (a, b, c) surface and in (d, e, f) cross section.


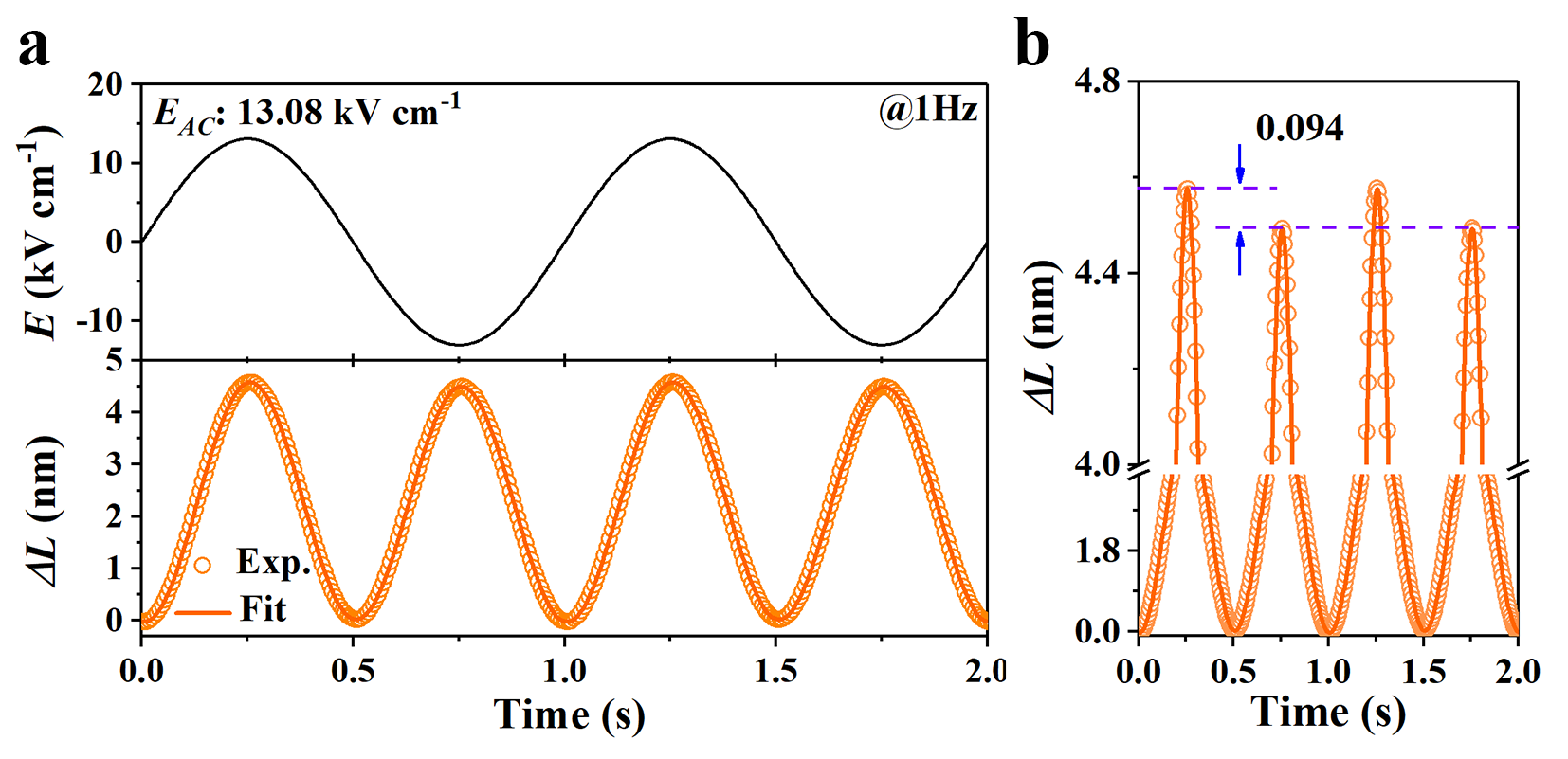


Figure S4. (a) The time-resolved electromechanical displacement under the electric field of *E*_AC_=13.08 kV cm^-1^ at 1 Hz for [100]-oriented SC-YSZ sample, and the measured thickness change *ΔL* in time was fitted by the Fourier sine series (orange line). (b) Expanded view of the *ΔL* in Figure S4a.


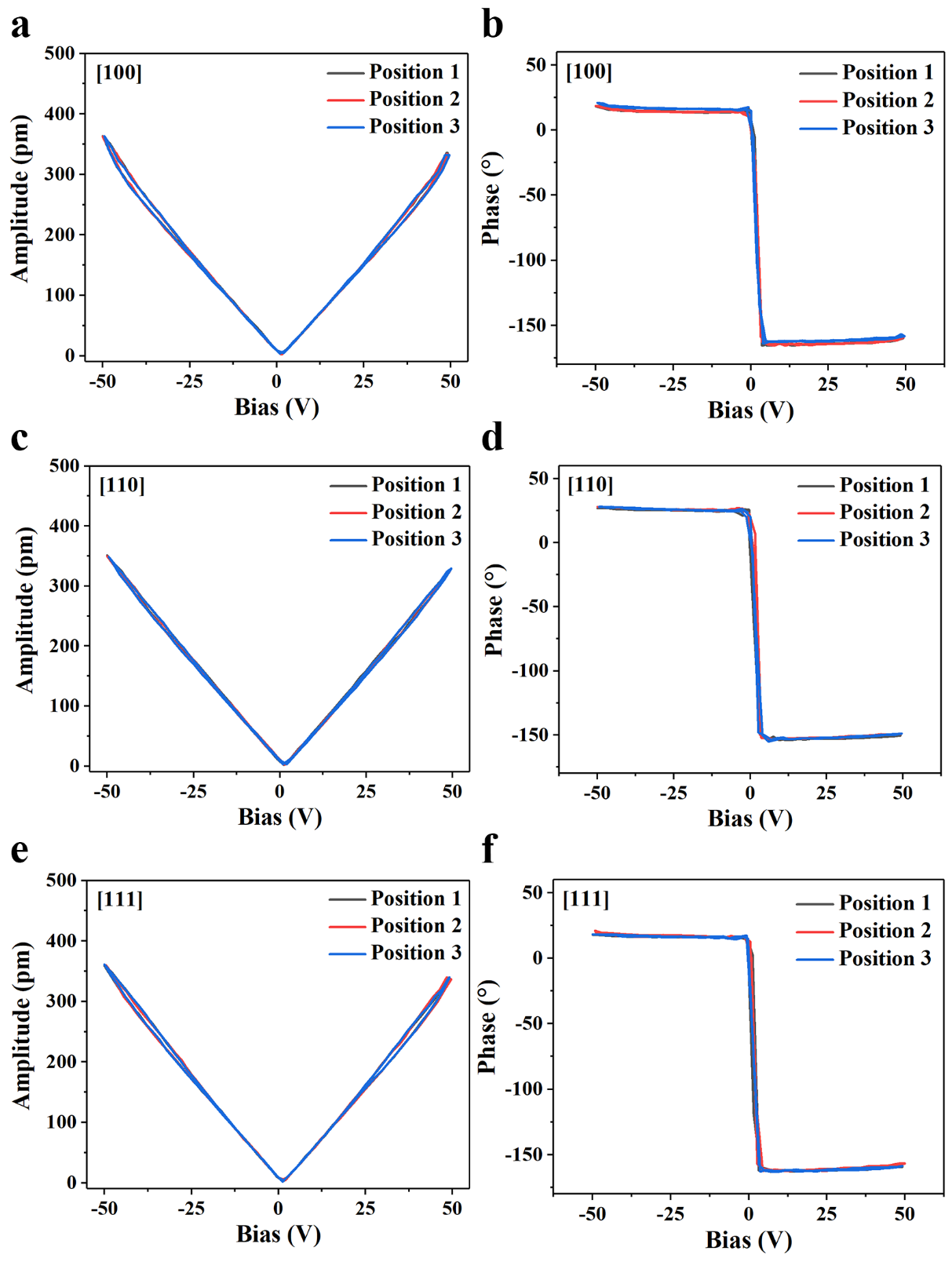


Figure S5. The amplitude and phase of the (a, b) [100]-, (c, d) [110]-, and (e, f) [111]-oriented SC-YSZ samples under different DC bias, and each sample was measured at 3 different positions on the top electrode.


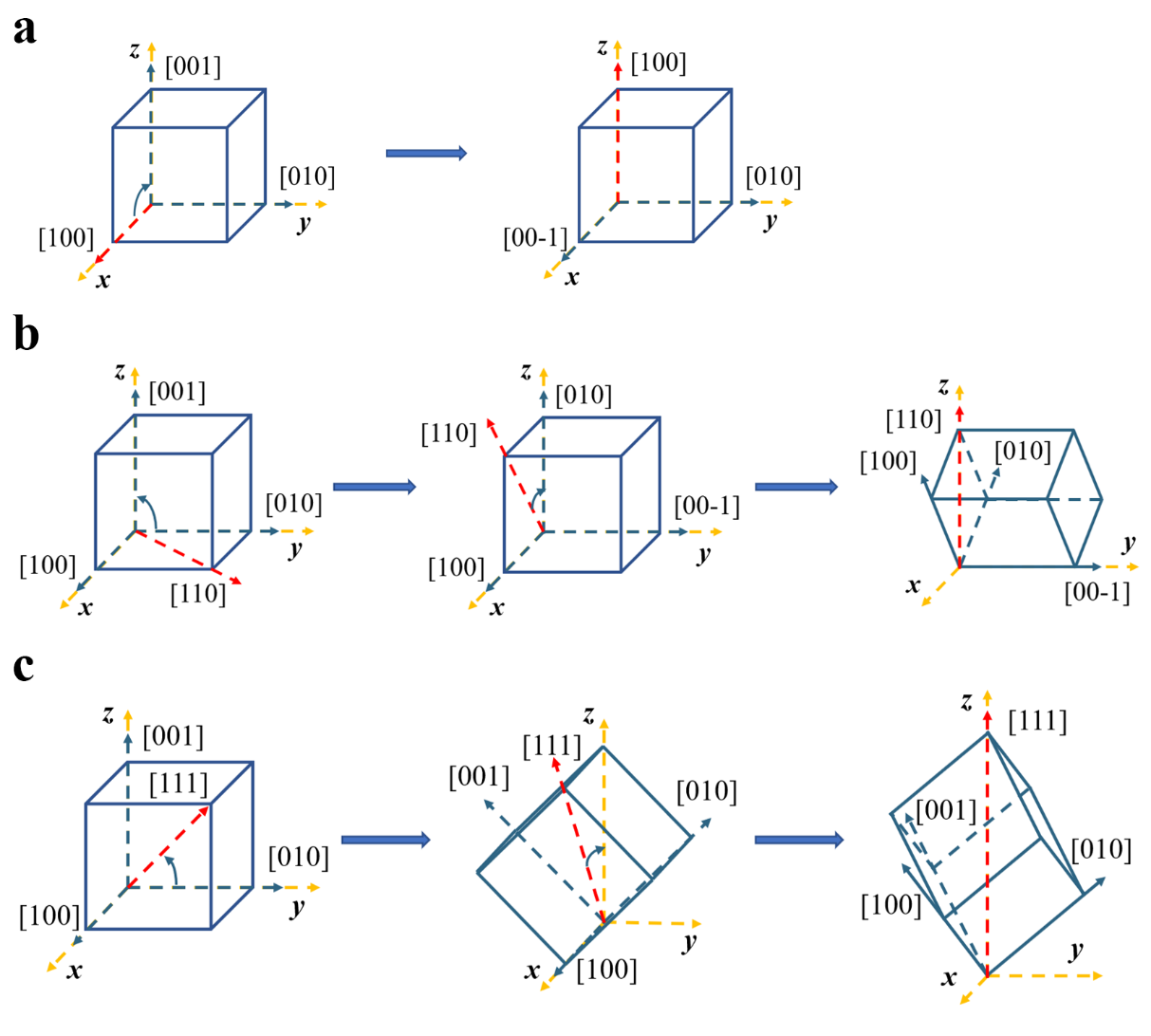


Figure S6. The rotation diagram from [001]-oriented SC-YSZ to global coordinate in (a) [100]-, (b) [110]-, and (c) [111]-oriented SC-YSZ samples.


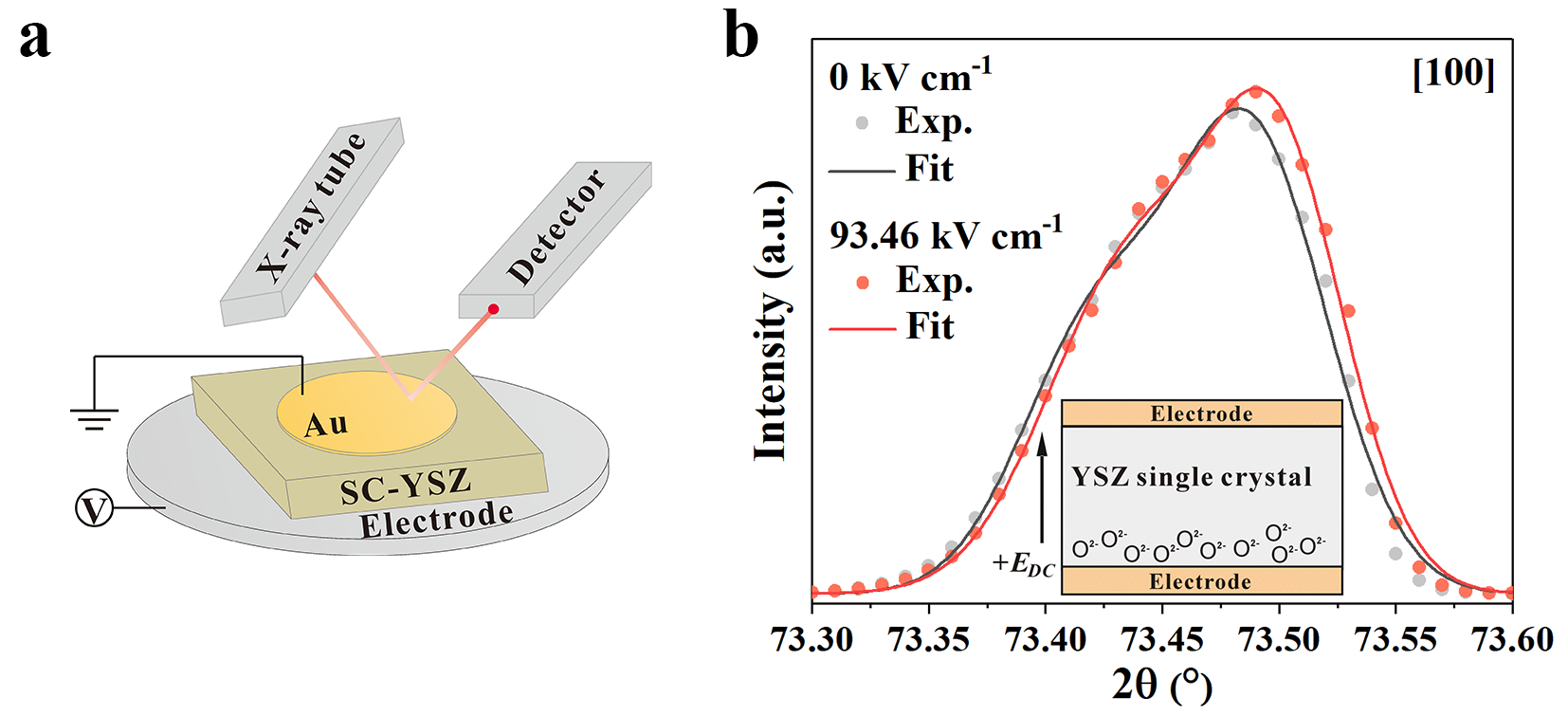


Figure S7. (a) The schematic diagram of the in-situ XRD experiment. (b) The in-situ XRD patterns of [100]-oriented SC-YSZ sample in absence (grey circle & black line) and in presence (orange circle & red line) of the electric field *E*_DC_. The inset describes the schematic for the electric field applied to SC-YSZ by applying *E*_DC_=1000 V.

Table S1. The pseudo-piezoelectric coefficients of the YSZ materials under electric field at 1 Hz.

| **Materials** | ***E*_DC_** (kV cm^-1^) | **Measured** | **Expected** (*E*_DC_=1 MV cm^-1^) |
| --- | --- | --- | --- |
|  |  | ***d*_33_** (pm V^-1^) | ***d*_33_** (pm V^-1^) |
| YSZ ceramic^[6]^ | 80.00 | 5.05 | 63.13 |
| YSZ polycrystalline film^[6]^ | 917.00 | 1000.00 | 1090.51 |
| [100] SC-YSZ | 13.08 | 125.01 | 9557.34 |
| [110] SC-YSZ | 13.08 | 38.07 | 2910.55 |
| [111] SC-YSZ | 13.08 | 13.47 | 1029.82 |

Notes: Since *d*_33_ and *E*_DC_ are in a linear relationship,^[6]^ "Expected" is the predicted value of *d*_33_ under *E*_DC_=1 MV cm^-1^.

**References**
